# A Comparison of Autistic Like Traits in the Relatives of Patients with Autism and Schizophrenia Spectrum Disorder 

**Published:** 2018-04

**Authors:** Zeynab Khanjani, Shahin Azmoodeh, Majid Mahmoudaliloo, Gholamreza Noorazar

**Affiliations:** 1 Department of Education and Psychology, Tabriz University, Tabriz, Iran.; 2 Department of Psychology, Urmia University, Urmia, Iran.; 3 Tabriz University of Medical Sciences, Tabriz, Iran.

**Keywords:** *Autistic Like Traits*, *Autism Spectrum Disorder*, *Relatives of Individuals with Autism*, *Relatives of Patients with Schizophrenia*, *Schizophrenia Spectrum Disorder*

## Abstract

**Objective:** This study aimed to identify autistic like traits in relatives of patients with schizophrenia and autism spectrum disorder.

**Method** **:** causal-comparative research design was utilized. Fifty individuals among the first degree relatives of patients with autism spectrum disorder and 50 individuals among the first degree relatives of patients with schizophrenia spectrum disorder were selected. Autistic-like traits were evaluated by Autism-Spectrum Quotient (AQ). Multivariate analysis of variance was used to compare the autistic like traits in two groups.

**Results:** First degree relatives of individuals with autism spectrum disorder got higher scores in deficiency of social skill, deficiency of communication, deficiency of attention, and attention to details. As well as they got lower scores in deficiency of imagination, in comparison to relatives of individuals with schizophrenia spectrum disorder.

**Conclusion: **Relatives of individuals with autism spectrum disorder compared to relatives of patients with schizophrenia spectrum disorder showed higher rates of autistic like traits. Only the exception was imagination subscale.

Most mental disorders have genetic component; though no specific genes known as the transmitter factor of specific mental illnesses. Surveys show that if parents are suffering from a disorders, the incidence of the disorder in their children is more than others. The term of autism was suggested primarily by Swiss psychiatrist Eugen Bleuler for defining the symptoms of schizophrenia (1). Leo Kanner used "autistic loneliness" in a number of children who were extremely intelligent, but were secluded and didn’t attend to others and insisted on uniformity (2). Then he called this state "early infantile autism". Asperger diagnosed milder form of autism in some individuals; nowadays we know it as Asperger's syndrome (3). 

In 2013, in DSM-5 all sub types including autism, Asperger and others were gathered in an umbrella term known as autism spectrum disorder (4). 

The prevalence of ASD in worldwide is about 1.0% (4), and it is raising yearly. The Prevalence of this disorder in Iran is about 1per 100 children. Some evidence shows that social and language defects in autism spectrum disorders are often seen hereditary. Twin studies confirmed that there is a genetic factor in autism spectrum disorder. Although several studies emphasized that there is a genetic component in autism spectrum disorder, responsible genes have not been identified. 

Autism Spectrum Disorder is a complex disorder in which a variety of different genes contribute to development and severity of symptoms. Now it seems that only 15% of autism spectrum disorder is associated with the genetic mutations (4).

There have been disagreements over whether Asperger's syndrome is inside the autism spectrum or not (5, 6, 7). Nowadays there is an agreement in this area and even in the DSM-5 this disorder is considered in the autism spectrum disorders. Diagnosis is according to the severity of symptoms, the high severity is related to autism and the low to Asperger and between individuals with Asperger and ordinary people are individuals with autistic like traits. These traits are similar to symptoms that patients with autism spectrum disorder experience, but in the milder form. These symptoms include some deficits in social skills, attention switching, communication, imagination and high attention to details. According to a study, the fathers and grandfathers of people with autism spectrum disorder compared with normal people are related more than two times with engineering (8). In another study results showed that autism was seen significantly more among families that were involved with physics, engineering and mathematics (9). In a meeting that was held on the occasion of autism, it was mentioned that the high rate of autism was seen in families of Albert Einstein, so having mild autistic like traits are not so bad and can cause improvements in the field of mathematics and engineering. Baron Cohen mentioned that the ability of individuals with these mild traits is because of their brains capability in concentrating on details or the parameters of the system (10). The twin studies conducted that heritability of autistic traits was very high (64% to 92% for different cut-off points) (11). Therefore, the possible existence of these traits in relatives of autism spectrum disorders is proposed.

Schizophrenia spectrum disorder includes positive symptoms such as delusion, hallucination, disorganized thinking (speech), and grossly disorganized or catatonic behavior and negative symptoms including loss of emotional expression, passivity, and lack of speech, pleasure, and socialization. Among the symptoms, loss of emotional expression and passivity are dominant in schizophrenia (4). Regarding the schizophrenia symptoms and high genetic predisposition to this disorder, presence of sub-threshold symptoms of schizophrenia in the first-degree relatives of these patients is probable. As there are some similarities in symptoms of schizophrenia and autistic like traits, the presence of these traits in relatives of patients with schizophrenia spectrum disorder is expected. 

Some studies about intergenerational transmission of sub-threshold autistic like traits in the general population have been done. Children from families in which both parents had sub-threshold autistic like traits showed a substantial shift in the distribution of their scores for impairment in reciprocal social behavior (12). Some researchers also studied the effects of familial risk factors and the place of birth on the risk of autism and found the highest risk of autism in families with a history of autism, Asperger's syndrome and other pervasive developmental disorders (PDDs) and concluded that genetic factors are implicated in autism (13). In another study autistic social impairment in the siblings of children with PPDs has been studied and results demonstrated the genetic predisposition in existence of this trait in relatives of these patients (14). In a study on autistic traits, the results showed that, patients with autism and schizophrenia spectrum disorders have deficiency in terms of interpersonal relationship (15). Some researchers studied autism dimensions in artists and scientists, the results showed there is no strong support for the relationship of scientific creativity to certain components of the autism spectrum disorder (16). In another study, 21 patients with autism and 21 patients with schizophrenia were investigated using AQ questionnaire, and results indicated that patients with autism showed deficits in social skills. Overlap was found between the two groups in attention to detail and imagination sub scales (17). A research showed that patients with autism spectrum disorder and schizotypal disorder have deficiency in interpersonal relationships (18). In another research the relationship between early autistic traits and psychosis in adulthood were studied, the results showed that autistic traits in childhood such as problems in talking and stereotypic and unusual habits can correlate with adult psychosis (19). A study showed that schizophrenia and autism are not related with each other (20). But another Study showed that these two disorders are tightly linked (21). The results of research are inconsistent. Therefore, this study aimed to investigate genetic contribution in autism spectrum disorders, as well as the possibility of autistic like traits existence in relatives of individuals with autism and schizophrenia spectrum disorders.

## Materials and Methods

This study was a causal-comparative research. 100 participants consist of first degree relative of individuals with autism spectrum disorder, aged between 7 and 16 years (n=50) from Tabriz autism center and first degree relatives of individuals with schizophrenia spectrum disorder, aged between 20 and 30 years (n=50) from Tabriz Razi hospital were selected. Participants completed autistic like traits questionnaires (AQ). Besides, in the present study GARS-2 was completed by mothers of patients with autism spectrum disorder to confirm the diagnosis of autism.

*Autistic like traits questionnaires (AQ)*

This questionnaire was used for measuring autism like traits in relatives of patients with autism and schizophrenia spectrum disorders. It’s a 50-item self-report questionnaire that assesses personal preferences and habits. This scale has five subscales, each composed of 10 items. Subscales include social skills, communication, imagination, attention switching, and attention to detail. For each item, participant's express agreement or disagreement on a four-point Likert type scale ranging from “definitely agree’” to “definitely disagree”. This scale was translated to Persian by Pouretemad et al, and is first introduced by Autism Research Center (ARC) in the UK. Baron - Cohen and et al suggest that as people with autism obtain high score on this questionnaire, it has high divergent validity (22). Hoekstra and colleagues showed that the internal consistency and test–retest reliability of this questionnaire is satisfactory (23). This questionnaire hasn’t been validated in Iran. Cronbach’s α for the present study is 0.73.

*GARS-2*

Gilliam Autism Rating Scale – second edition (GARS-2) is completed by teachers and parents and is used to diagnose autism in individuals aged 3 years to 22 years and in estimating the severity of the child's disorder (24). Items in GARS-2 are based on the definition of autism adopted by DSM-IV-TR. This instrument was normed on a representative sample of 1,107 people with autism from 48 states across the USA. The assessment consists of 42 clearly stated items that describe the characteristic behaviors of individuals with autism. The items are grouped into three subtests that include Stereotyped Behaviors, Communication, and Social Interaction. Each of these subtests contains of 14 items. each question is answered in a four-point Likert scale; scored from 0 to 3 for “Never”, “rarely”, “sometimes”, “very much” respectively. Cronbach's α for this scale for stereotyped behaviors is 0.9, for communication 0.89, for social interaction 0.93, and for entire questionnaire is 0.96.

## Results

In the present study, for comparing autistic like traits in two groups, multivariate analysis of variance was used.

Table 1 shows descriptive statistics with mean, standard deviation, minimum and maximum for autistic like traits in relatives of patients with autism and schizophrenia spectrum disorder. Results showed the average of autistic like trait in relatives of individuals with autism spectrum disorder is 27.9 changing between 19 (Min) to 41 (Max). In relatives of individuals with schizophrenia spectrum disorder the mean score of the autistic trait is 21.7 changing between 17 (Min) to 27 (Max).

The mean deficiency in social skills in relatives of patients with autism spectrum disorder is 6 and in relatives of patients with schizophrenia spectrum disorder is 4.8 (table 2). The mean score of deficiency in communication skill in relatives of patients with autism spectrum disorder is 5.67 and in schizophrenia spectrum disorder is 4.27. The mean score of imagination subscale in relatives of patients with autism spectrum disorder is 4.37 and in relatives of patients with schizophrenia spectrum disorder is 4.9. The mean score of deficiency in attention switching subscale in relatives of patients with autism spectrum disorder is 5.83 and in relatives of patients with schizophrenia spectrum disorder is 4.37. The mean score of attention to detail in patients with autism spectrum disorder is 6.03 and in relatives of patients with schizophrenia spectrum disorder is 3.4. To determine significant differences between autistic like traits in relatives of patients with schizophrenia and autism spectrum disorder multivariate analysis of variance (MANOVA) was used.

As shown in table 3 Wilks’ Lambda with the amount of 0.446 in level of 0.001 was significant. relatives of patients with schizophrenia and autism have significant differences in at least one of the dependent variables (social skills, Communication, imagination, attention switching, and attention to detail). Therefore, each of the dependent variables in two groups was studied.

**Table 1:** Descriptive Statistics Such as Mean, Standard Deviation, Minimum and Maximum for Autistic Like Traits (Total) in Relatives of Patients with Autism and Schizophrenia Spectrum Disorder

**Relatives**	**N**	**µ**	**S**	**Min**	**Max**
Autism	100	27.9000	4.73687	19.00	41.00
Schizophrenia	100	21.7333	2.66437	17.00	27.00

**Table 2:** The Mean and Standard Deviation of Autistic Like Traits in Two Groups of Autism and Schizophrenia Spectrum Disorder Relatives

**Autistic Like Traits**	**N**	**Relatives of Patients with Autism**		**Relatives of Patients with Schizophrenia**	
		**µ**	**S**	**µ**	**S**
					
social skills	200	6.00	1.86	4.80	1.94
communication	200	5.67	2.38	4.27	1.11
imagination	200	4.37	2.22	4.90	1.18
attention switching	200	5.83	1.46	4.37	2.03
attention to detail	200	6.03	1.33	3.40	2.14

According to means in table 2 and significances in table 4, Deficiency in social skills in relatives of autism patients with mean score of 6 is significantly higher than the relatives of schizophrenia with mean score of 4.8 (p <0.05). 

Deficiency in communication in relatives of autism patients with mean score of 5.67 is significantly higher than the relatives of schizophrenia with a mean score of 4.27 (p <0.01). 

Deficiency in imagination in relatives of autism patients with mean score of 4.37 is a little higher in comparison to relatives of schizophrenia patients with mean score of 4.9.

 Deficiency in attention switching in relatives of autism patients with mean score of 5.83 is significantly higher than the relatives of schizophrenia with mean score of 4.37 (p <0.01). 

Attention to details in relatives of autism patients with mean score of 6.03 is significantly higher than the relatives of schizophrenia with mean score of 3.4 (p <0.01).

## Discussion

The findings of this study showed that relatives of patients with autism spectrum disorder had higher scores than relatives of patients with schizophrenia spectrum disorder in four components of autistic like traits including deficiency in social skills, communication, attention switching, and high attention to details are in higher level. defect in imagination was similar in both groups and didn’t show significant difference. Perhaps we should refer to some theoreticians (5, 6), proposed that the highest point of spectrum is autism and the lowest is Asperger. between Asperger's disorders and ordinary people there are people with autistic like traits. These traits are similar to symptoms of patients with autism and include deficits in social skills, communication and imagination, attention to details, and attention switching. According to a report, fathers and grandfathers of persons with autism spectrum disorder are active in engineering about 2 times more than persons without (8). In another study performed by Baron-Cohen et al the results showed that autism spectrum disorder is significantly higher among children of families involved in physics, engineering and mathematics (9). A survey conducted on twins showed that heritability of autistic like traits was about 64% to 92% (11). In other study personality traits of the relatives of autistic probands were studied and concluded that particular personality traits may compact in the family members of autistic probands and in addition some of these traits may cause autism liability (25). In studying of autistic like traits in the general population, it was found that genes that affect autistic like traits are similar in boys and girls (26). Intensity and a lower incidence of autistic traits in females may be the result of the early effects of high environmental sensitivity. Austin proved high scores on the AQ questionnaire indicating weakness in social skills, communication, attention switching and high attention to detail (27). Piven and et al showed that high levels of deficiency in social skills and communication are seen in relatives of persons with autism spectrum disorder (28). Also Constantino and Todd demonstrated deficiency in social relationships in children's of parents with autism (26). According to Leo kanner (2), the main feature of autism spectrum disorder is inability to build relationships with other people and situations in normal manner; this deficiency causes high "autistic loneliness". This loneliness is seen significantly in areas such as language, behavior, intellectual growth, and cognitive and social relationships. Evidence shows that social and language deficits and mental problems are often seen in family members of persons with autism. Studying siblings of patients with autism showed that the prevalence rate of autism in siblings is 2 to 14 percent, much higher than the prevalence rate in the general population that is 0.05 to 0.2 percent (4). Due to the possible role of genetic in development of this disorder, presence of autistic like traits in relatives of patients with autism spectrum disorder is likely. Relatives of patients with autism spectrum disorder prefer to be alone and have weak social skills and have problems in coping easily with social situations. These people aren’t eager to communicate and speak in public. Being weak in attention switching in these people results in preference of doing tasks in old manner or they are weak in performing more than one task simultaneously. Attention to detail is high in these people so that they consider to some details that are not important for others. Present study showed that these two groups are similar in imagination subscale and there isn’t significant difference between them, but that does not mean that these two groups don’t have problems in imagination and are similar to ordinary people by considering that they have significant differences in comparison with normal people. Imagination is weak in these people in a way that they cannot understand the feelings of others and put themselves in the shoes of another person. In contrast to the findings of this study about imagination, most studies report that imagination in schizophrenia can be very high. Evidence shows that these patients tend to create a lot of false memories (29). False memories may reflect a situation in which a person was supposed to do a task but in fact have not done it, and this imagination can become a false memory (30). Perhaps the difference between present study’s findings with others in imagination term is related to the meaning of this word; for example, the study that has been done by Lee et al considered false memories as imagination. A high score on the AQ questionnaire means that a person is weak in imagination that Austin demonstrated it in his research (27).

**Table 3:** Multivariate Covariance Analysis (MANOVA) for the Dependent Variables

	**Value**	**F**	**Hypothesis df**	**Error df**	**Sig**
Pillai’s Trace	0.554	13.40	5.000	194.000	0.000
Wilks’ Lambda	0.446	13.40	5.000	194.000	0.000
Hotelling’s Trace	1.241	13.40	5.000	194.000	0.000
Roy’s Largest Root	1.241	13.40	5.000	194.000	0.000

**Table 4:** Multivariate Analysis of Variance (MANOVA) for the Dependent Variables

**Source**	**Dependent ** **V** **ariable**	**Sum of Squares**	**df**	**Mean of Square**	**F**	**S** **ig**
group	social skills	21.600	1	21.600	6.000	0.017
	Communication	29.400	1	29.400	8.503	0.005
	Imagination	4.267	1	4.267	1.347	0.250
	attention switching	32.267	1	32.267	10.332	0.002
	attention to detail	104.017	1	104.017	32.758	0.000

## Limitation

In the present study, we used self-report questionnaire and the collected data may be the subject of response bias. This research was conducted on first-degree relatives, so in generalizing of these results to the second and third degree relatives should be carried out with caution. By using other research methods such as interviews and observations in the future studies, the possible bias will be decreased. If a similar study performed on the second and third-degree relatives, the results will be comparable with the findings of the present investigation.

## Conclusion

As expected, relatives of individuals with autism spectrum disorder compared to schizophrenia spectrum disorder relatives showed high rates of autistic like traits subscales. Only the exception was imagination subscale. It is recommended that by referring the relatives of patients with autism and schizophrenia spectrum disorders that show autistic like traits to health centers, future problems can be prevented, and by apprising them about the risk factors we can convince them to use health care services until the symptoms will be moderated. Also it is recommended that by giving awareness about the high proportion of genetic in these disorders, an important step can be preventing consanguineous marriage. 
